# Phosphorylation of Ribosomal Protein RPS6 Integrates Light Signals and Circadian Clock Signals

**DOI:** 10.3389/fpls.2017.02210

**Published:** 2018-01-19

**Authors:** Ramya Enganti, Sung Ki Cho, Jody D. Toperzer, Ricardo A. Urquidi-Camacho, Ozkan S. Cakir, Alexandria P. Ray, Paul E. Abraham, Robert L. Hettich, Albrecht G. von Arnim

**Affiliations:** ^1^Department of Biochemistry, Cellular and Molecular Biology, The University of Tennessee, Knoxville, TN, United States; ^2^Graduate School of Genome Science and Technology, The University of Tennessee, Knoxville, TN, United States; ^3^Chemical Sciences Division, Oak Ridge National Laboratory, Oak Ridge, TN, United States

**Keywords:** translation, circadian clock, RPS6, phosphorylation, diurnal, *Arabidopsis*, eS6

## Abstract

The translation of mRNA into protein is tightly regulated by the light environment as well as by the circadian clock. Although changes in translational efficiency have been well documented at the level of mRNA-ribosome loading, the underlying mechanisms are unclear. The reversible phosphorylation of RIBOSOMAL PROTEIN OF THE SMALL SUBUNIT 6 (RPS6) has been known for 40 years, but the biochemical significance of this event remains unclear to this day. Here, we confirm using a clock-deficient strain of *Arabidopsis thaliana* that RPS6 phosphorylation (RPS6-P) is controlled by the diel light-dark cycle with a peak during the day. Strikingly, when wild-type, clock-enabled, seedlings that have been entrained to a light-dark cycle are placed under free-running conditions, the circadian clock drives a cycle of RPS6-P with an opposite phase, peaking during the subjective night. We show that in wild-type seedlings under a light-dark cycle, the incoherent light and clock signals are integrated by the plant to cause an oscillation in RPS6-P with a reduced amplitude with a peak during the day. Sucrose can stimulate RPS6-P, as seen when sucrose in the medium masks the light response of etiolated seedlings. However, the diel cycles of RPS6-P are observed in the presence of 1% sucrose and in its absence. Sucrose at a high concentration of 3% appears to interfere with the robust integration of light and clock signals at the level of RPS6-P. Finally, we addressed whether RPS6-P occurs uniformly in polysomes, non-polysomal ribosomes and their subunits, and non-ribosomal protein. It is the polysomal RPS6 whose phosphorylation is most highly stimulated by light and repressed by darkness. These data exemplify a striking case of contrasting biochemical regulation between clock signals and light signals. Although the physiological significance of RPS6-P remains unknown, our data provide a mechanistic basis for the future understanding of this enigmatic event.

## Introduction

The translation of mRNA into protein is regulated by light and darkness in conjunction with the circadian clock. The transition of dark-grown *Arabidopsis thaliana* seedlings to light is accompanied by a broad stimulation of ribosome loading for a large fraction of the transcriptome, while transfer of light-grown seedlings to darkness results in the translational repression of numerous mRNAs (Tang et al., 2003; Juntawong and Bailey-Serres, 2012; Liu et al., 2012, 2013). Accordingly, in both seedlings and vegetative rosettes, the fraction of ribosomes that are associated with mRNA in the form of polyribosomes fluctuates in a diel fashion while the total number of ribosomes remains constant (Piques et al., 2009; Pal et al., 2013; Missra et al., 2015). Over the diel day-night cycle, mRNAs fall into three major groups, those lacking a discernible cycle of ribosome loading, those with peak ribosome loading during the day, and another group with peak ribosome loading during the night. Ribosome loading of mRNAs is also under the control of the circadian clock, as indicated by broad changes in the phase and amplitude of diel translation in a clock-deficient strain. The mRNAs for ribosomal proteins are among those with the most pronounced and coordinated changes in diel ribosome loading (Missra et al., 2015; Mills et al., 2017). These findings indicate that signals from the light environment and signals from the circadian clock must be integrated as they converge onto the translation apparatus. The point of this integration is unknown.

The phosphoprotein RPS6 (RIBOSOMAL PROTEIN OF THE SMALL SUBUNIT6, eS6) (Ban et al., 2014) is a well known target of environmental stimuli, given that heat and hypoxia and a low energy balance trigger dephosphorylation of RPS6 (Scharf and Nover, 1982; Williams et al., 2003; Nukarinen et al., 2016) while auxin, cold, and sucrose boost its phosphorylation (Beltran-Pena et al., 2002; Williams et al., 2003; Turck et al., 2004; Dobrenel et al., 2016). RPS6, which is encoded by two paralogous genes in *Arabidopsis* (Creff et al., 2010), is phosphorylated on up to five (RPS6B) or seven (RPS6A) serine or threonine residues in the carboxyl-terminal tail of the protein (Durek et al., 2010; Turkina et al., 2011; Boex-Fontvieille et al., 2013). RPS6 phosphorylation (RPS6-P) occurs in all organisms where it has been examined, from yeast to plants and humans, but despite intensive study the biochemical consequence of RPS6 phosphorylation for translation is essentially unknown (Chauvin et al., 2014; Meyuhas, 2015; Yerlikaya et al., 2016). Early studies suggesting an effect of RPS6-P on ribosome activity in HeLa cells have been questioned (Duncan and McConkey, 1982; Tas and Martini, 1987). Mice engineered to harbor only a non-phosphorylatable RPS6 have numerous organ-level and health abnormalities (Ruvinsky et al., 2009; Meyuhas, 2015). A report suggested dramatically increased translation activity in phospho-negative tissue culture cells derived from such mice (Ruvinsky et al., 2005), which may be connected to reduced translational fidelity (Wittenberg et al., 2016). However, no mRNA-specific defects have been identified. RPS6-P has been linked to ribosome biogenesis in the mouse (Chauvin et al., 2014), and a role for RPS6, but not yet RPS6-P, in ribosome biogenesis has been proposed in *Arabidopsis* (Kim et al., 2014; Son et al., 2015). Taken together, the biochemical consequence of RPS6 phosphorylation is largely unknown. The lack of any well characterized function notwithstanding, RPS6-P is widely regarded as a bioreporter for TOR kinase activity (Biever et al., 2015; Dobrenel et al., 2016). Important for this study, in *Arabidopsis*, phosphorylation of RPS6 is regulated in a diel fashion with an elevated level during the light period. Serine 237 and serine 240 are two residues that are highly conserved and most commonly found to be phosphorylated in *Arabidopsis* and maize (Williams et al., 2003; Turkina et al., 2011; Boex-Fontvieille et al., 2013; Choudhary et al., 2015; Nukarinen et al., 2016).

We asked whether the phosphorylation of RPS6 may be one of the cellular events that integrate the effect of light and clock signals on the translation apparatus. Does RPS6-P serve as a biomarker for the convergence of light and clock signals on the translation apparatus? Here we show using a pair of new, phospho-specific antibodies that RPS6-P cycles with a peak during the day under light-dark cycle illumination, in keeping with an acute stimulation of phosphorylation by various qualities of light. These cycles are particularly robust in a clock-deficient strain. In stark contrast, in a clock-enabled strain grown under constant illumination RPS6 phosphorylation on serine 237 also cycles, but with a peak during the subjective night. In combination, in clock-enabled plants under a light-dark cycle, RPS6-P cycles in a fashion suggesting that the circadian clock tempers the amplitude of light-driven RPS6-P cycles. Contrary to expectations, supplemental sucrose had only minor effects and did not confound the integration of light and clock signals. Finally, we show that the dynamics of RPS6 phosphorylation during the dark-to-light transition after dawn is most pronounced on polyribosomes, i.e., actively translating ribosomes, while monosomes and free subunits experience more subtle changes in the phosphorylation status of RPS6.

## Materials and Methods

### Plant Material and Growth Conditions

Strains included Columbia (Col-0) and the CCA1-overexpressor (CCA1-ox) (Wang and Tobin, 1998). Seeds were sterilized and stratified at 4°C for 2 days. They were sown onto solid ½ strength Murashige and Skoog medium without sucrose or supplemented with 1 or 3% sucrose and grown under long day conditions (16 h light/8 h dark) for 12 days at a constant temperature of 22°C and constant light intensity of 70 μE⋅m^-2^⋅s^-1^.

### Light Treatments

Light emitting diodes housed in shielded cabinets (Stacey et al., 2000) were used to supply blue, red and far-red light. Seedlings were grown in the dark for 4 days followed by exposure to blue light at 20 μE⋅m^-2^⋅s^-1^ or red light at 50 μE⋅m^-2^⋅s^-1^ or far-red light intensity of 322 μE⋅m^-2^⋅s^-1^. For white light treatment, the plates were kept in a growth chamber with a light intensity of 70 μE⋅m^-2^⋅s^-1^. For the constant light treatment, the seedlings were first grown under long-day conditions for 11 days and then shifted into a chamber with constant light intensity. Similarly, for the constant dark treatment, seedlings were grown under long-day for 11 days and then shifted into a dark room.

### Stress Treatments

For heat treatment, wild-type Col seedlings were grown under long day for 12 days and shifted into a lighted chamber at 37°C. Control seedlings were collected from plates at 22°C. For cold treatment, seedlings were grown under long day for 12 days at 22°C and shifted into a lighted chamber at 4°C. Control seedlings were collected from plates at 22°C.

### Antibody Generation

Antibodies against RPS6 were generated (Agrisera, Umeå, Sweden) by conjugating the peptide CDKRISQEVSGDALGEE to keyhole limpet hemocyanin (KLH) and injecting it into two rabbits. Phospho-specific antibodies against S237-P and S240-P were generated by conjugating the peptides KKR(S*^P^*)RLSSAAC for S237 and KKRSRL(S*^P^*)SAAC for S240 to KLH and injecting it into two rabbits. Four rounds of immunization were performed over the course of 82 days. An ELISA test was performed on day 68 to determine the titer of the antibodies and the rabbits were bled on day 93. To detect RPS6, the serum from the final bleed was used in subsequent experiments. The phospho-antibodies were purified by first depleting the antisera against immobilized non-phosphorylated peptide followed by positive selection against immobilized phosphorylated peptide. The RPS6 antibody detected unphosphorylated His-tagged RPS6 produced in *E. coli*, but the phospho-specific antibodies did not. In addition, the signal detected by these antibodies in plant extract declined when phosphatase inhibitors were omitted from the extraction buffer (Supplementary Figures 1A,B). Therefore, the antibodies are specific to the phosphorylated form of RPS6.

### SDS–PAGE and Immunoblotting

Total proteins were extracted from Col-0 seedlings in an extraction buffer 100 mM KPO_4_ pH 7.8, 1 mM EDTA, 1% Triton X-100, 10% Glycerol, 1 mM DTT, protease inhibitors (Pierce #88666) and phosphatase inhibitors (Pierce #88667) and then separated on 15% SDS–PAGE gels. The proteins were transferred to polyvinylidene difluoride membranes (PVDF, Millipore) and transferred proteins were visualized by staining with Ponceau S. Membranes were blocked with non-fat dry milk and then probed with either anti-RPS6 rabbit polyclonal antiserum (dilution 1:2500) or anti -RPS6 S237-P rabbit polyclonal IgG (dilution 1:5000) or anti-RPS6 S240-P rabbit polyclonal IgG (dilution 1:5000). Goat anti-rabbit IgG coupled to horse radish peroxidase, 1:2500 (Vector Labs Inc.) as a secondary antibody was detected using WesternBright Quantum ECL chemiluminescence reagents (Advansta).

### Western Blot Quantification

The western blots were quantified using ImageJ. The intensity of each band was calculated individually and the intensities of the signals for S237-P and S240-P were then normalized to RPS6 to control for differences in protein loading per gel lane. The S237/RPS6 and S240/RPS6 ratios over one time course were median centered for all the replicates and the averages were plotted as a XY graph using GraphPad Prism software. The period length and phase of the diel cycles were calculated with BioDare2 (Zielinski et al., 2014), a diurnal data analysis software (MESA algorithm). Cosine curves with the parameters from BioDare2 were drawn into selected graphs.

### Protein Digestion and Phosphopeptide Enrichment

WT *Arabidopsis* seedlings were grown on media without sucrose, entrained with LD and shifted to LL or kept in LD. The seedlings were collected at ZT0 and ZT12 and were ground under liquid nitrogen using a mortar and pestle. For each sample, 500 mg of ground tissue was suspended in SDS lysis buffer (2% SDS and 10 mM DTT in 100 mM ammonium bicarbonate) supplemented with Halt Phosphatase Inhibitor Cocktail (Thermo Fischer Scientific), boiled for 5 min, sonically disrupted (30% amplitude, 10 s pulse with 10 s rest, 2 min total pulse time) and boiled for an additional 5 min. Crude protein extract was pre-cleared via centrifugation, and quantified by BCA assay (Pierce Biotechnology). Proteins were alkylated with iodoacetamide (30 mM) and incubated in the dark at room temperature for 15 min. Protein was extracted via methanol/chloroform/water precipitation and protein pellets were washed twice with methanol. Dried proteins pellets were resuspended in 1 mL of 8 M urea, incubated at room temperature for 1 hr. Samples were digested via addition of two aliquots of sequencing-grade trypsin (Promega, 1:50 [w:w]) at two different sample dilutions, 4 M urea (overnight) and subsequent 2 M urea (5 h). Following digestion, samples were adjusted to 1% formic acid and desalted using solid-phase C18 extraction cartridges (Sep-Pak Plus Short, Waters), and lyophilized. For each sample, 2 mg of desalted peptides were then processed using the High-Select Fe-NTA Phosphopeptide Enrichment Kit (Thermo Fischer Scientific) protocol.

### LC-MS/MS

All spectra were acquired on a QExactive Plus (Thermo Fischer Scientific) coupled to an Easy-nLC 1200 (Thermo Fischer Scientific) ultrahigh pressure liquid chromatography (UHPLC) pump. Peptides were separated on an in-house packed 75 μm inner diameter column containing 30 cm of Kinetix C18 resin (1.7 μm, 100 Å, Phenomenex) with a gradient consisting of 5–22% (80% CAN, 0.1% FA) over 120 min at 200 nL/min. Data acquisition was managed by XCalibur version 4.0. Mass spectra were acquired in a data-dependent mode. MS1 spectra were collected at 70,000 resolution, with an automated gain control (AGC) target of 1E6, and a max injection time of 25 ms. The 15 most intense ions were selected for MS/MS. Precursors were filtered according to charge state and monoisotopic assignment. Previously interrogated precursors were excluded using a dynamic window of 45.0 s ± 8 ppm. The MS/MS precursors were isolated with a quadrupole mass filter set to a width of 1.6 *m/z*. Precursors were fragmented by high-energy collision dissociation (HCD) at a normalized collision energy (NCE) of 30%. The MS/MS spectra were collected at 30,000 resolution, with an AGC target of 1E6 and a max injection time of 50 ms.

### Peptide Identification and Protein Inference

All experimental MS/MS spectra were compared to theoretical tryptic peptide sequences generated from a FASTA database containing the full protein complement of the *A. thaliana* TAIR 10 database, appended with common contaminant proteins (Mellacheruvu et al., 2013). A decoy database, consisting of the reversed sequences of the target database, was appended to discern the false-discovery rate (FDR). Assignment of MS/MS spectra was performed using MSFragger algorithm (Kong et al., 2017). The MSFragger closed search was configured to derive fully tryptic peptides with the following parameters: parent mass tolerance of 20 ppm, a fragment mass tolerance of 20 ppm, a static modification (C+57.0214 Da), a dynamic modification corresponding to an oxidation (M+15.9949 Da) of methionine, and a dynamic modification corresponding to potential phosphorylation events (STY+79.9663). The max number of dynamic mods allowed were 3. Post-processing was performed with Peptide (Keller et al., 2002) and Protein Prophet (Nesvizhskii et al., 2003) and data was filtered to a 1% FDR at the peptide-spectrum match, peptide and protein level. For the phosphorylated peptides of interest, we manually extracted ion chromatograms with Qual Browser (Thermo Fischer Scientific) using a mass tolerance of 10 ppm and determined the area of the curve to assign a relative abundance value per peptide.

### Polysome Profiling

Plant tissue was ground in liquid nitrogen and extracted in polysome isolation buffer (200 mM Tris-HCl pH 8.4, 25 mM MgCl_2_, 50 mM KCl, 1% deoxycholic acid and 2% polyoxyethylene 10 tridecyl ether). Sucrose gradients were prepared by layering 1.68 ml, 3.34 ml, 3.34 ml, and 1.68 ml each of 50% sucrose, 38.4% sucrose, 26.6% sucrose, and 15% sucrose, respectively. After addition of each gradient layer, the centrifuge tube was frozen at -80°C for 1 h. On the day before use, the gradients were thawed overnight without shaking at 4°C. Plant extracts (1 ml) were loaded on top of a 10 ml 15–50% sucrose gradient and centrifuged at 35,000 rpm for 3.5 h (Beckmann Coulter SW 41Ti). The gradient was fractionated into 12 equal fractions after the absorbance at 254 nm had been recorded to determine the RNA profile. Samples from the fractions were then separated on SDS–PAGE gels followed by immunoblotting to determine RPS6 protein levels and phosphorylation levels in the samples. Equal volumes of sample from each fraction were loaded onto the gels.

To determine the ratio of polysomes and monosomes, RNA absorption profiles as well as blank profiles from a gradient with buffer only were imported into R (version 3.4.0) (R Core Team, 2017). The blank gradient was used as a baseline and subtracted from the RNA profiles. Peaks and troughs were assigned manually to the various ribosomal complexes. Areas underneath the monosomal and polysomal peaks were calculated using the trapz function in the pracma package (version 2.0.7) (Borchers, 2017). The monosome area was defined as the area underneath the 40S, 60S, and 80S peaks.

### Accession Numbers

RPS6A At4g31700, RPS6B At5g10360, CCA1 At2g46830, LHY At1g01060, PRR5 At5g24470, PRR7 At5g02810, PRR9 At2g46790. The phosphoproteome data were deposited at ProteomeXchange (Deutsch et al., 2017) with identifier PXD008125.

## Results

### Dynamics of RPS6 Phosphorylation in Wild-Type Seedlings

**FIGURE 1:**
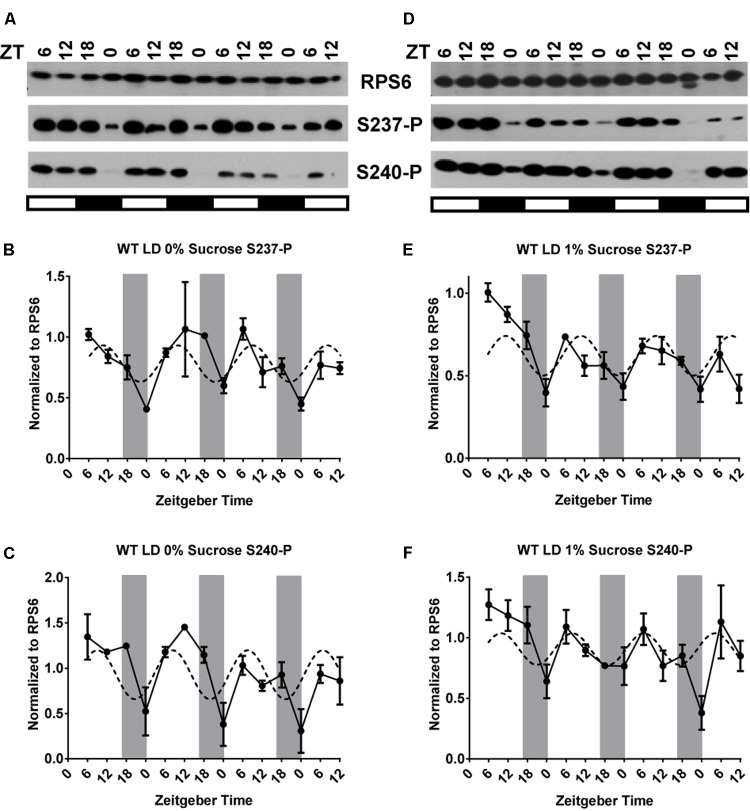
In wild-type seedlings under a light dark cycle, light-driven and clock-driven cycles combine into a low-amplitude cycle with peak phosphorylation during the day. Seedlings of wild-type *Arabidopsis* were entrained in a light-dark cycle for 12 days, then grown under the same conditions for another three and a half days and scored for phosphorylation of RPS6 (ribosomal protein eS6) every 6 h. Seedlings were grown on medium lacking sucrose **(A–C)** or containing 1% sucrose **(D–F)**. Phosphorylation at S237 (S237-P) and at S240 (S240-P) was quantified using phosphospecific antibodies. **(A,D)** Representative immunoblots for S237-P and S240-P. The total amount of RPS6 was determined with an antibody against an amino-terminal peptide of RPS6. Time is given in hours of zeitgeber time (ZT) after lights-on on Day 12. The white and black bars indicate light and dark conditions, respectively. **(B,C,E,F)** Immunoblot signals for S237-P and S240-P were quantified using ImageJ software and normalized against total RPS6 signals from the same time point. Each data series was median-centered and data from multiple replicates were averaged. Error bars show the standard error of the mean from *n* = 2 replicates for 0% sucrose and *n* = 3 for 1% sucrose, grown at different times, which were immunoblotted once or twice each. A sine curve was fitted to the data as described in Section “Materials and Methods” (dashed line). The dark gray bars indicate the daily dark period.

In an effort to characterize the diel phosphorylation dynamics of RPS6, we first present the cycle of RPS6-P in 12-day-old seedlings of wild-type under a long-day light-dark cycle (LD, 16 h light/8 h dark) in the absence of exogenous sucrose. Phosphorylation of both S237-P and S240-P cycled reproducibly over 3 days with a 24 h period with a peak during the day and a trough around dawn (ZT0) (**Figures 1A–C**). The patterns for S237-P and S240-P were similar, indicating coordinated phosphorylation after dawn and dephosphorylation during the night. In these, as well as all other experiments described below, the total level of RPS6 protein did not change significantly, in keeping with the long half-life of ribosomal proteins (Li et al., 2017). The peak phosphorylation around ZT11 is consistent with prior data (Turkina et al., 2011; Boex-Fontvieille et al., 2013). To further confirm the day peak of RPS6-P, we performed the experiment with a higher time resolution by collecting samples every 2 h over a 24 h period. As seen in the previous experiment, RPS6-P was lowest late in the night with a peak during the day (Supplementary Figures 2A–C).

### In the Absence of a Functional Clock, RPS6-P Cycles with a Robust Peak during the Day

To test whether the cycle of phosphorylation is driven substantially by the circadian clock, we repeated the experiment in the clock-deficient CCA1-overexpressing strain (CCA1-ox) grown under a LD cycle. The CCA1-ox strain was chosen as a clock-deficient strain (Wang and Tobin, 1998) because it balances a near normal growth habit with a near arrhythmic phenotype. Under light-dark cycles, the transcript cycles of 10 core clock genes are either phase-delayed, or are derepressed or repressed, causing a reduced amplitude, and in constant light several core clock transcripts are largely arrhythmic (Matsushika et al., 2002; Missra et al., 2015). For comparison, the *cca1 lhy* mutant retains residual rhythms (Mizoguchi et al., 2002), and the *prr5 prr7 prr9* triple mutant, while substantially arrhythmic, has a strong shade avoidance phenotype (Matsushika et al., 2000; Nakamichi et al., 2005). Finally, the metabolome of CCA1-ox is more similar to wild-type than that of *prr5 prr7 prr9* (Nakamichi et al., 2009), and CCA1-ox depletes its starch reserves at a similar pace as wild-type, other than *cca1 lhy* and *prr5 prr7 prr9* (Ruts et al., 2012). Under our growth conditions, the leaf size of CCA1-ox was the same as wild-type. The CCA1-ox strain has a mildly elongated hypocotyl at the seedling stage. It also flowers late, which is of little concern to our data from 12-day-old seedlings.

**FIGURE 2:**
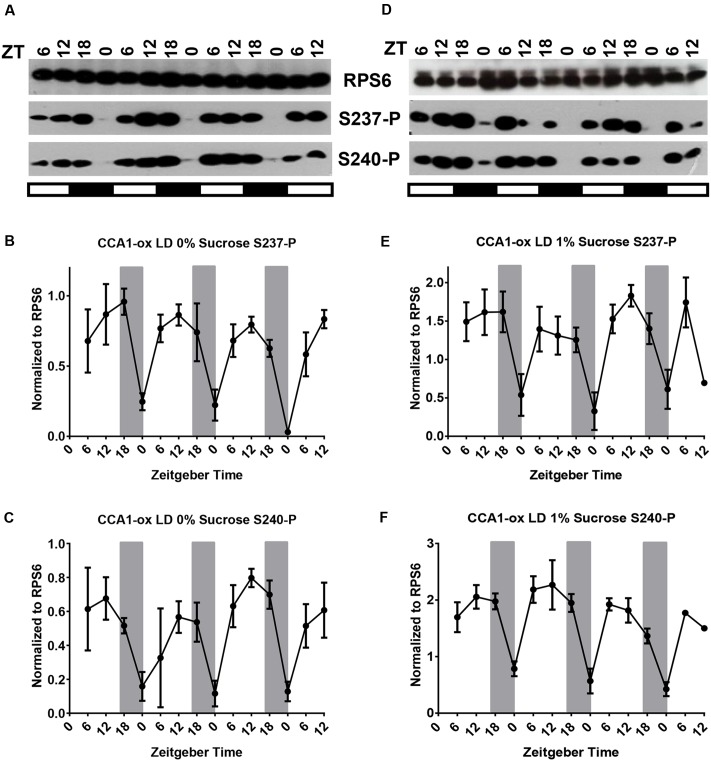
A long-day light-dark cycle drives cycles of RPS6 phosphorylation with a peak during the day. 12-day-old seedlings of *Arabidopsis* clock-deficient plants (CCA1-ox) were scored for phosphorylation of RPS6 (ribosomal protein eS6) every 6 h for another three and a half days. Seedlings were grown in a long-day light-dark cycle on medium lacking sucrose **(A–C)** or containing 1% sucrose **(D–F)**. **(A,D)** Representative immunoblots for S237-P and S240-P. **(B,C,E,F)** Immunoblot signals for S237-P and S240-P were quantified and normalized against total RPS6 signals from the same time point. Error bars show the standard error of the mean from *n* = 2 replicates for 0% sucrose and *n* = 3 for 1% sucrose, grown at different times, which were immunoblotted once or twice each. For details see legend to **Figure 1**.

For both S237-P and S240-P, RPS6-P cycled with a peak late during the day in CCA1-ox (**Figures 2A–C**) (**Table 1**). Evidently, the circadian clock is not required for robust cycles, suggesting that the cycles are driven by the changes in the light environment. Averaged over multiple biological replicates, the amplitude of the cycles was larger in the clock-deficient strain (*p* = 0.011 by Student’s *t*-test), implying that the clock may even attenuate the amplitude of the phosphorylation cycles.

**Table 1:** Summary of RPS6-P patterns in WT (clock-enabled) and CCA1-ox (clock-deficient) strains under LD, LL, and DD conditions.

Genotype	WT	CCA1-ox	WT	CCA1-ox	WT	CCA1-ox
Clock	Enabled	Deficient	Enabled	Deficient	Enabled	Deficient
Light	LD	LD	LL	LL	DD	DD
0% Sucrose	Day Peak (S237 and S240)	Day Peak (S237 and S240)	Night Peak (S237)	Acyclic	Negligible	Negligible
1% Sucrose	Day Peak (S237 and S240)	Day Peak (S237 and S240)	Night Peak (S237)	Acyclic	Negligible	Negligible
3% Sucrose	Variable Peak (S237)	Day Peak (S237 and S240)	Night Peak (S237)	Acyclic	Acyclic	Acyclic
	Erratic (S240)					


### Under the Control of the Circadian Clock, RPS6-P Cycles with a Peak during the Subjective Night

**FIGURE 3:**
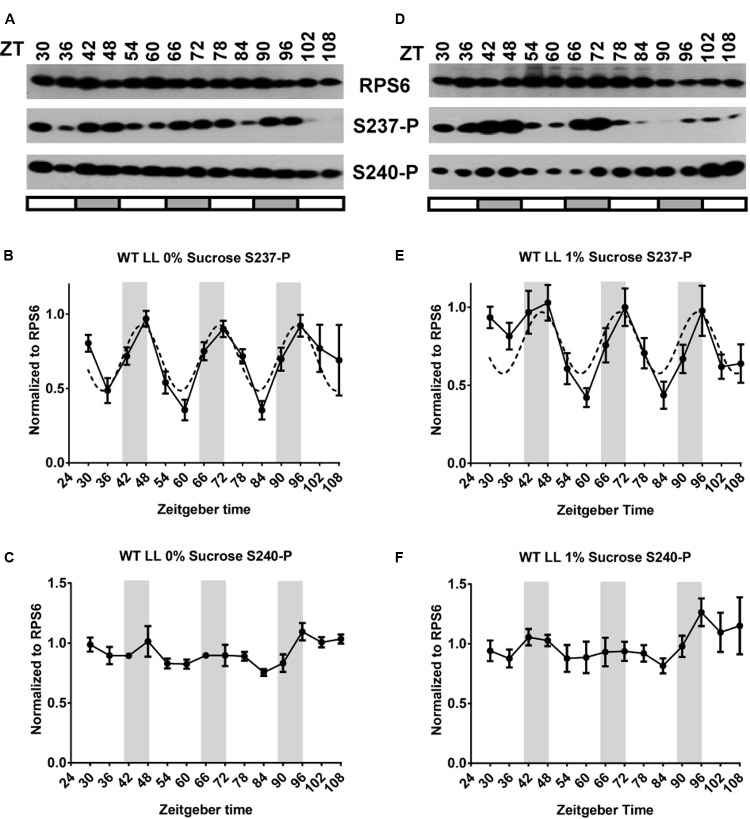
The circadian clock drives cycles of RPS6 phosphorylation with a peak during the night. Seedlings of wild-type *Arabidopsis* were entrained in a light-dark cycle for 11 days, then shifted to continuous light for another four and a half days. Sampling began at ZT30 on the second day in continuous light. Seedlings were grown on medium lacking sucrose **(A–C)** or containing 1% sucrose **(D–F)**. **(A,D)** Representative immunoblots for S237-P and S240-P and total RPS6. **(B,C,E,F)** Immunoblot signals for S237-P and S240-P were quantified and normalized against total RPS6 signals from the same time point, followed by median-centering each data series and averaging of the multiple replicates. Error bars show the standard error of the mean from *n* = 4 replicates for 0% sucrose and *n* = 5 for 1% sucrose.

To examine whether and how the circadian clock regulates RPS6 phosphorylation, wild-type seedlings were entrained with long-day (LD) light-dark cycles and then shifted to continuous light (LL) for 4 days. Sampling started on Day 2 at subjective noon, ZT30 (**Figures 3A–C**). Under these conditions, RPS6-P cycled as well, but, surprisingly, the phosphorylation levels peaked during the subjective night. It is noteworthy that only S237-P cycled with a clear ‘night’ peak while S240-P did not. An experiment with higher time resolution under LL confirmed that S237-P peaked around subjective dawn whereas S240-P did not cycle (Supplementary Figures 2D–F). For WT under LD conditions, the period was calculated at 23.2 ± 0.4 h with a phase of ZT12.0 ± 1.1 for S237-P and, similarly, a period of 23.2 ± 0.8 h with a phase of ZT13.0 ± 0.2 for S240-P. Under LL the period was 24.2 ± 1.4 h for S237-P with a phase of ZT0.1 ± 1.2 for S237-P. These fitted curves clearly indicate that the clock-driven cycle is shifted by about 12 h compared to the cycle under long-day conditions.

Of note, in an earlier phosphoproteomic study of *Arabidopsis* grown under LL but on 3% sucrose, cyclic phosphorylation on RPS6 was mapped to S240 and S241, as well as S237 (Choudhary et al., 2015). Therefore, we compared the dynamic phosphorylation of serines in RPS6 by mass spectrometry under our growth conditions for both LD and LL (**Table 2**). Under LD, S231, S237, S240 and S241 had elevated phosphorylation during the day, at ZT12 compared to ZT0. Also consistent with our western blot data, under LL, S237-P and S241-P were elevated at ZT0 compared to ZT12. However, the mass spectrometry data also indicated elevated phosphorylation at S240 at ZT0 over ZT12, although the fold change was lower for S240-P compared to S237-P and S241-P. The lack of a cycle for S240-P in LL in our western blot might be due to the reduced amplitude of the cycle as well as the likely possibility that the S240-P epitope is masked by additional cyclical phosphorylation of S241.

**Table 2:** Phosphorylated RPS6 peptides detected by LC-MS/MS under LD and LL.

			LD			LL		
				
Protein	Peptide	Site	Peak	Fold change	*p*-value	Peak	Fold change	*p*-value
RPS6A	SRLSSAAAKPSVTA	S240	ZT12	4.29	0.003	ZT0	1.81	0.083
RPS6A	LSSAAAKPSVTA	S241	ZT12	3.14	0.005	ZT0	2.94	0.006
RPS6A	KGENDLPGLTDTEKPR	T127	ZT12	0.88	0.599	ZT0	1.17	0.469
RPS6A	SRLSSAAAKPSVTA	S237, S240	ZT12	2.90	0.020	ZT0	3.51	0.019
RPS6A/B	SESLAKK	S231	ZT12	2.07	<0.001	ZT0	1.21	0.371
RPS6A/B	LVTPLTLQR	T185	ZT12	2.06	0.088	–	–	–
RPS6B	SRLSSAPAKPVAA	S240	ZT12	2.24	0.003	ZT0	1.54	0.166
RPS6B	SRLSSAPAKPVAA	S240, S237/S241	ZT12	3.39	0.002	ZT0	3.98	0.008


*In each peptide sequence, the phosphorylated serine (S) is underlined. For one peptide it was not possible to distinguish between phosphorylation on S237 or S241. Significance of fold-changes between high and low values was tested by *t*-test.*

**FIGURE 4:**
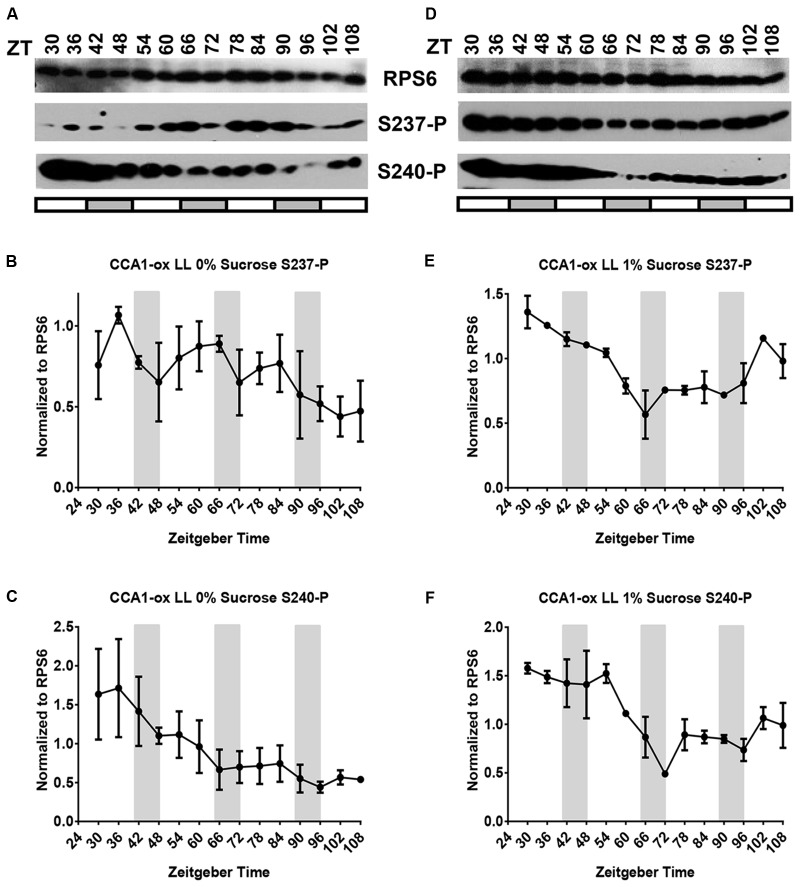
The CCA1-ox strain cannot sustain cycles of RPS6 phosphorylation if grown in continuous light. Seedlings of clock-deficient *Arabidopsis* (CCA1-ox) were entrained in a light-dark cycle for 11 days, then shifted to continuous light for another four and a half days. Sampling began at ZT30 on the second day in continuous light. Seedlings were grown on medium lacking sucrose **(A–C)** or containing 1% sucrose **(D–F)**. **(A,D)** Representative immunoblots for S237-P and S240-P and total RPS6. **(B,C,E,F)** Immunoblot signals for S237-P and S240-P were quantified and normalized against total RPS6 signals from the same time point, followed by median-centering each data series and averaging of the multiple replicates. Error bars show the standard error of the mean from *n* = 2 replicates.

To confirm that the night peak of RPS6 S237-P under free-running conditions was driven by the circadian clock, we repeated the LL experiment in the clock-deficient CCA1-ox strain (**Figures 4A–C**). In the absence of a clock the phosphorylation of both S237-P and S240-P was generally elevated, but it drifted without any periodicity and no reproducible pattern. Conversely, when CCA1-ox was transferred to continuous darkness (cDD), RPS6-P dropped during Day 1 and was nearly undetectable between Day 2 and Day 4; the same was true in wild-type (Supplementary Figure 3, left panels). In summary, while light-dark cycles can drive a robust oscillation of RPS6-P, cycles of RPS6-P can also be driven by the circadian clock, but with a diametrically opposite phase. The clock-driven cycles require light, given that they do not persist in continuous darkness.

### Integration of Light-Dark Signals and Circadian Clock Signals

For many cycling transcripts whose phasing has been measured carefully under both clock-driven and light-driven conditions, the phase of the clock-driven cycle coincides closely with the phase of the light-driven cycle; a common exception being that the clock allows the transcript to anticipate the dawn. In contrast, a phase difference of around 12 h between a light-dark driven cycle and a clock-drive cycle as seen for RPS6-P is unusual.

As we consider how the contrasting clock signals and light signals are integrated in a wild-type plant under a long-day light-dark cycle there are four theoretical scenarios. If light and clock pathways were integrated via a logic gate, then (i) light eclipses the clock effect, or (ii) clock eclipses light. (iii) Light and clock cycles may also superimpose additively, which would predict that light-dark and clock signals for S237-P would nearly cancel each other out; and (iv) light and clock signals may be quantitatively integrated, for example according to the ‘product rule’ of independent effects. RPS6-P continued to cycle in wild-type under LD, but at an elevated level and, at least in most of our experiments, with a reduced amplitude (**Figure 1**). Therefore, the integration of light and clock signals is best represented by scenario (iv). Light signals dominate, but the sharp drop in phosphorylation toward the end of night can be attenuated by the clock signal. Scenario (ii) and, arguably, (i) and (iii) are not as easily reconciled with the data.

### Control of RPS6-P by Light

**FIGURE 5:**
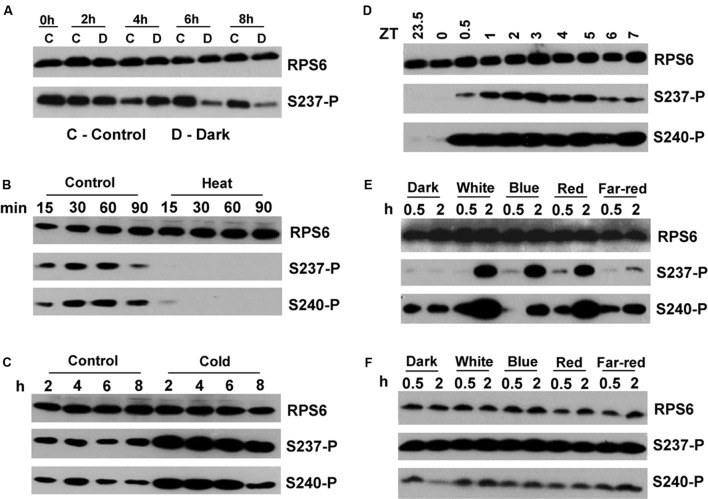
Acute responses of RPS6-phosphorylation to light and darkness, heat and cold. All seedlings are wild-type and 12-days-old. Experiments were replicated twice with similar results. **(A)** Seedlings grown in a LD cycle on 1% sucrose were shifted to darkness at ZT 4 for the indicated times. Control seedlings were maintained in the light. **(B)** Seedlings grown in a LD cycle on 0% sucrose were subjected to 37°C heat in the light for the indicated times. **(C)** Seedlings grown in a LD cycle on 0% sucrose were subjected to 4°C in the light for the indicated times. **(D)** Seedlings grown in a LD cycle on 1% sucrose were transferred to white light for the indicated times. **(E)** Etiolated seedlings germinated in darkness without sucrose were exposed to the indicated light qualities for 0.5 or 2 h. **(F)** Same as **(E)** but in the presence of 1% sucrose. Note that levels of phosphorylation were elevated even in darkness. Absolute signal strength should not be compared with that in other panels because exposure times for the immunoblots differed.

Because the cycle of RPS6-P is dominated by the light environment, we then asked, how rapidly does RPS6-P decline when entrained, light-grown seedlings are exposed to darkness in the middle of the day? RPS6-P was rather stable at this time, and only declined reproducibly after about 4h to 6h (**Figure 5A**), in sharp contrast to the very rapid decline of RPS6-P, well within 30 min, after exposure to 37°C heat stress and a rapid increase within 2 h after transfer from ambient to 4°C (**Figures 5B,C**). We also timed the rise in RPS6-P after dawn in the morning, and found a rapid increase within 30 min (**Figure 5D**). Given that RPS6-P also does not decline rapidly after the regular dusk at ZT16 (**Figure 1**), these data suggest, that, while RPS6-P can change rapidly, its decline in response to darkness is buffered against rapid changes in illumination.

When *Arabidopsis* seedlings were germinated in darkness in the absence of sucrose, exposure to blue, red or far-red light caused a rapid increase in phosphorylation of RPS6 (**Figure 5E**). Given the immediacy of the response at a time when the etiolated seedlings have not turned green yet, the phosphorylation cannot be attributed to the production of photosynthate. Therefore, we conclude that RPS6-P is induced by the photoreceptors as a part of photomorphogenetic development. However, in the presence of 1% sucrose in the growth medium, RPS6 was strongly phosphorylated in dark-grown seedlings, which substantially masked any induction of RPS6-P by light (**Figure 5F**).

### Effect of Sucrose on Cycles of RPS6-P in Light-Grown Seedlings

Given that etiolated seedlings responded to sucrose with RPS6-P and given the stimulatory effect of sucrose on RPS6-P observed previously (Dobrenel et al., 2016), we suspected that supplemental sucrose might interfere with the diel cycles of RPS6-P that are driven by the coordinated action of light-dark cycles and the circadian clock. However, as shown in panels D–F of **Figures 1** through **4**, and summarized in Supplementary Figure 4 where data from all 3 days are overlaid onto a single 24-h time interval, sucrose at 1% had no dramatic effect on the pattern of RPS6-P.

**FIGURE 6:**
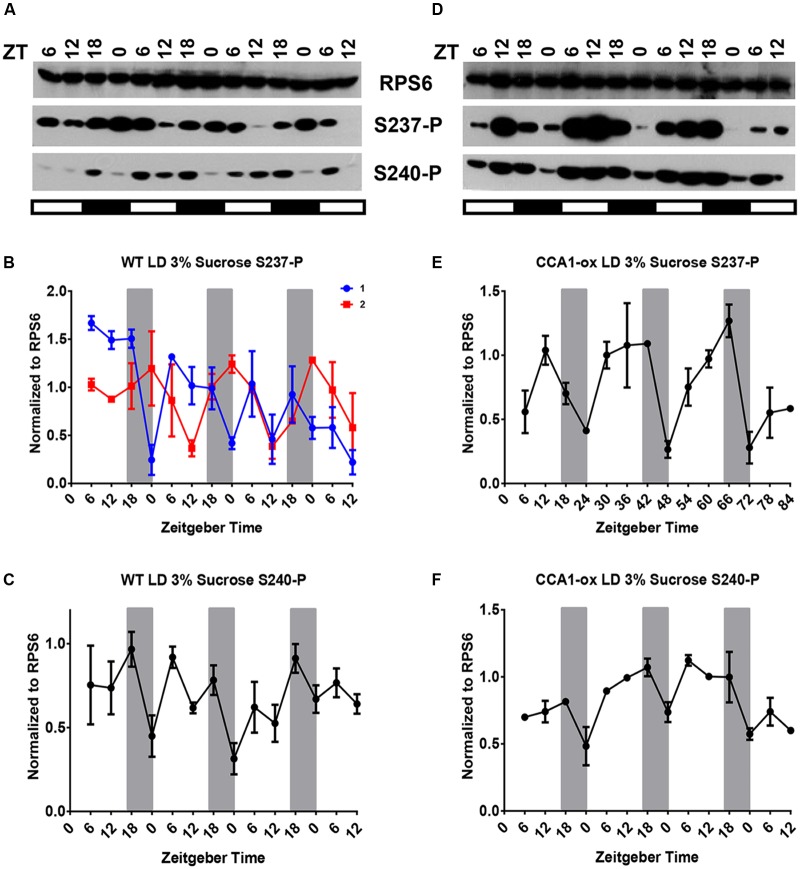
RPS6-P at 3% sucrose under long-day conditions. **(A–C)** Wild-type. **(D–F)** CCA1-ox. Experiments were performed at least twice at different times. For details see legend to **Figure 1**.

**FIGURE 7:**
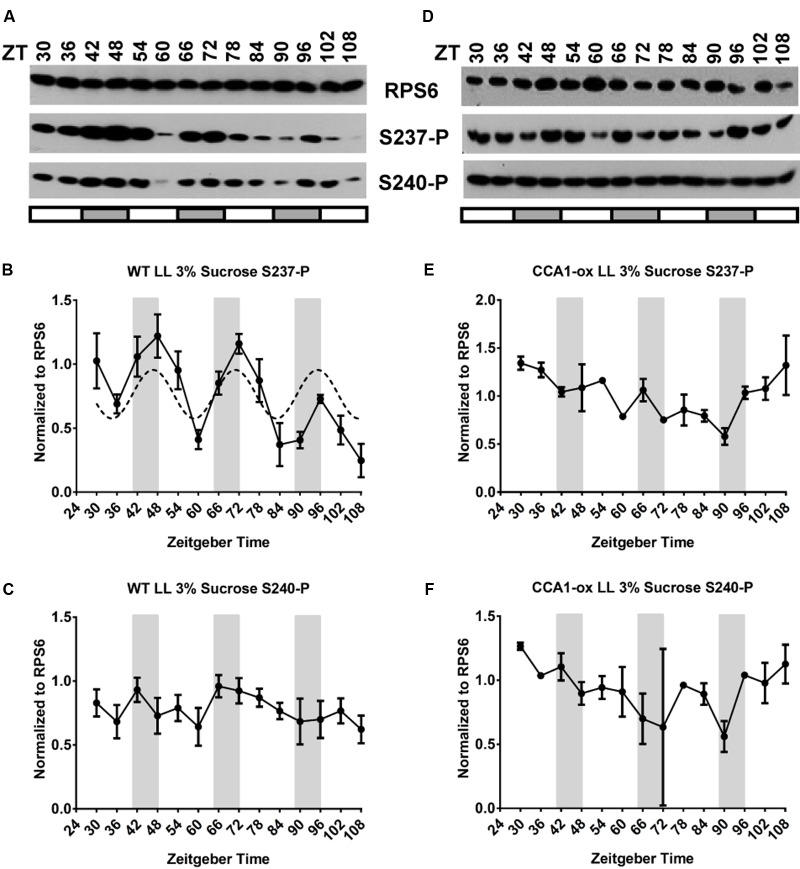
RPS6-P at 3% sucrose under continuous light. **(A–C)** Wild-type. **(D–F)** CCA1-ox. Experiments were performed at least twice at different times. For details see legend to **Figure 3**.

At 3% sucrose, the results depended strongly on the light conditions. In CCA1-ox in a LD cycle, the strong amplitude of the daytime peaks of S237-P were substantially maintained (**Figures 6D–F**), as were the equivalent peaks during subjective night in WT in LL (**Figures 7A–C**), although the cycles of S240-P were weak (CCA1-ox in LD) or indiscernible (WT in LL) at 3% sucrose. As expected, 3% sucrose also did not affect the broadly drifting RPS6-P in clock-deficient LL conditions (**Figures 7D–F**). However, in clock-enabled wild-type under LD the integrated cycles were retained at 1% sucrose (**Figures 1D–F**), but they were disturbed at 3% sucrose (**Figures 6A–C**). This disturbance manifested itself in two interesting ways. First, for S237-P, in one LD cycle experiment we observed a night-peak in the cycle of RPS6 S237-P during each of 3 days (**Figure 6B**), suggesting that in this exceptional experiment the clock effect overruled the light-dark effect; however, when the experiment was replicated, the regular day-peak was detected; each experiment comprised two separate samples of seedlings. Second, for S240-P the cycles under 3% sucrose followed a zig-zag pattern, reminiscent of a 12 h period, and very different from the typical 24 h period or the drifting pattern seen in clock-deficient plants under LL; this result was reproducible in both biological replicates (**Figure 6C**). These results suggest that the integration of light and clock signals is disturbed by a high concentration of sucrose.

Finally, in constant darkness, 3% sucrose caused a clear elevation in S237-P and S240-P over the minimal level observed in 0% or 1% sucrose (Supplementary Figure 3, right panels). This was seen in both clock-enabled wild-type and clock-deficient CCA1-ox; of note, S237-P and S240-P were poorly correlated and no 24 h period could be discerned.

In an attempt to discern subtle sucrose effects, we overlaid data from all 3 days onto a single 24-h time interval (Supplementary Figure 4). Taken together, supplementation of plants with sucrose did not dramatically affect the purely light driven (Supplementary Figures 4A,B) or purely clock driven cycles of RPS6-P (Supplementary Figure 4C for S237-P, S240-P had a weak clock driven cycle). Sucrose at 3% did, however, mask the preeminence of the light signal in driving a day-peak of RPS6 phosphorylation in wild-type seedlings in a LD cycle (Supplementary Figures 4G,H). These results suggest that high concentrations of sucrose disturb the integration of light signals and clock signals in this system.

### Representation of RPS6-P in Polysomes

**FIGURE 8:**
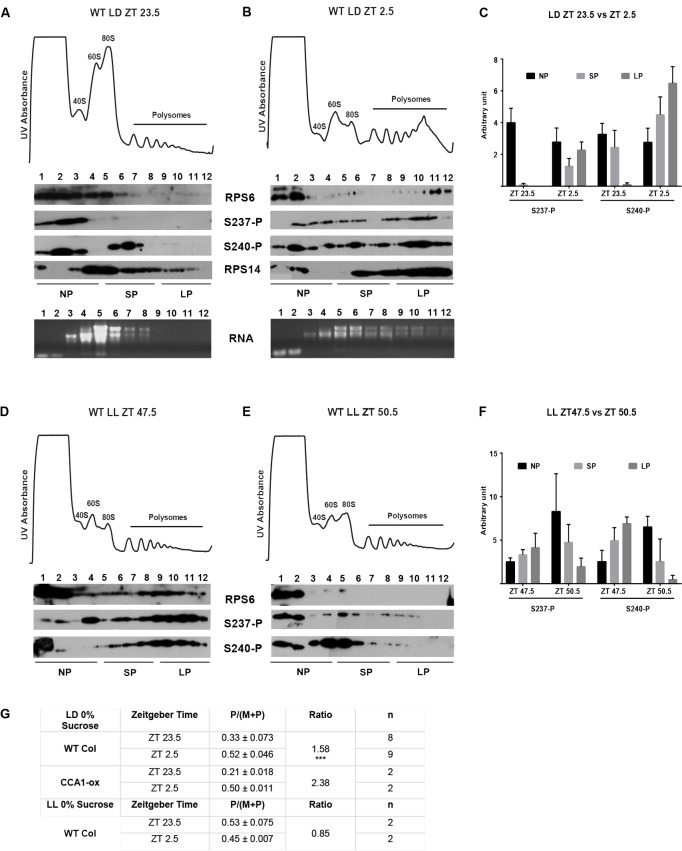
RPS6-P in polysomes. Twelve-day old wild-type seedlings were grown under the indicated light conditions (0% sucrose), and harvested at ZT23.5 (end of night) and ZT2.5 (morning) followed by sucrose gradient fractionation of total cell extracts. In the UV absorption profiles 40S, 60S, 80S ribosomes as well as polysomes are labeled. Equal volumes of each gradient fraction were probed for S237-P and S240-P and for total RPS6. **(A–C)** Wild-type seedlings grown in a long-day light-dark cycle and harvested at ZT23.5 **(A)** and ZT2.5 **(B)**. As controls, the fractions were additionally probed for RPS14 and RNA extracted from the fractions was run on a gel to show the distribution of the ribosomal RNA. Note that for the RPS6 antibody, it appears that exposure of RPS6 to the high sucrose concentration in the gradient results in a drop in affinity to the antibody when the samples are analyzed. **(C)** Immunoblot signals for S237-P and S240-P were quantified using ImageJ and were pooled into bins for non-polysomal material (fractions 1–4), small polysomes (fractions 5–8, including monosomes), and large polysomes (fractions 9–12). Data in **(C)** are from 6 biological replicates. **(D–F)** Same as **(A–C)**, but seedlings were shifted to continuous light on day 11 at ZT0 and harvested at the equivalent times (end of subjective night, subjective morning). **(G)** Table displaying the polysome/(monosome + polysome) (P/(M + P)) ratios and the ratio of ZT 23.5 to ZT 2.5 under LD and ZT 47.5 to ZT 50.5 under LL. Standard deviation was calculated for the P/(M + P) ratios and ^∗∗∗^ indicates *p* < 0.001 in a *t*-test.

In HeLa cells, phosphorylated RPS6 is found in polysomes as well as in non-polysomal ribosomes (Duncan and McConkey, 1982). We next tested whether the distribution of *Arabidopsis* RPS6-P between non-polysomal, small polysomal, and large polysomal fractions is affected by light, and whether S237 and S240 are always phosphorylated in a coordinated manner. Because RPS6 was phosphorylated most rapidly upon the dark-to-light transition (Supplementary Figure 2B), we focused on the time interval between end of night (ZT23.5) and mid-morning (ZT2.5). During this time interval light increases polysome loading by about 1.6-fold (**Figure 8G**). For the target site S237, RPS6 was essentially unphosphorylated at the end of night in monosomes, in small polysomes (1–4 ribosomes per mRNA) as well as in large polysomes (more than four ribosomes per mRNA); residual phosphorylation was observed mostly in fractions 1–2, containing non-ribosomal RPS6 protein and fraction 3, 40S subunits. S237 became heavily phosphorylated within 3 h (**Figures 8A–C**). The rise in polysomal RPS6-P greatly exceeded the approximately 60% rise in the fraction of polysomal ribosomes during the dark-to-light transition (**Figure 8G**). For S240, the pattern was similar, except that at the end of night small polysomes and monosomes retained high levels of phosphorylation (**Figures 8A–C**). These results indicate that the phosphorylation status of polysomal RPS6 is more dynamic than that of non-polysomal RPS6, suggesting that it is the polysomal RPS6 that is preferentially subject to phosphorylation and dephosphorylation.

For comparison, in wild-type seedlings grown in LL, RPS6 was still heavily phosphorylated at the end of the subjective night (**Figures 8D–F**), and this phosphorylation declined during the next 3 h into the subjective morning, in keeping with the decline in phosphorylation previously seen in total protein extracts (**Figure 3**), and in keeping with a downward trend in polysome loading (**Figure 8G**). As seen under LD cycle conditions, polysomal phosphorylation showed a more robust change than the change in total polysome loading. In both LD and LL, S240 tended to retain a higher level of phosphorylation than S237, for example in the non-polysomal fractions before dawn in LD, and after dawn in LL.

For comparison, when seedlings were treated with a 2 h 37°C heat shock followed by recovery for 2 h, phosphorylation of RPS6 declined rapidly in all ribosomal fractions, followed by a recovery. Again, S240 appeared to attract higher levels of phosphorylation than S237, especially during recovery. These changes in phosphorylation were rapid and mirrored those in the total polysome loading (Supplementary Figure 5). These data suggest that while S237-P and S240-P are commonly correlated, they can be partially uncoupled under some conditions.

In all polysome gradients, we noticed that a portion of RPS6 remained in the low molecular weight fraction at the top of the gradient where it was not associated with 40S ribosomes (non-ribosomal portion), as indicated by the fact that this fraction contained no detectable 18S rRNA and little RPS14 protein (**Figures 8A,B**). The non-ribosomal RPS6 was usually phosphorylated, but because its phosphorylation was somewhat variable we decided to focus on the small polysomal and large polysomal fractions of RPS6.

## Discussion

### Phase Shifting of a Clock-Driven Cycle by the Light-Dark Environment

Many plant responses are subject to diel cycles by the circadian clock, as seen in a clock-enabled wild-type under free-running conditions (LL), but also respond to light and darkness, as seen in a clock-deficient strain in LD. In wild-type plants under LD, when the clock is functional and entrained, these two signals are intertwined to drive a composite cycle. As an illustrative example for an approximately additive interaction, see the overlay of an acute light response at ZT1 and a broader clock-driven peak around ZT5 for the clock transcript *PRR9* (Matsushika et al., 2002; Jones et al., 2012). As another prior example, growth of the *Arabidopsis* hypocotyl peaks around dusk, both in wild-type grown in LL and also in the clock-deficient *elf3* strain in LD, but it peaks late at night in wild-type under a light-dark cycle; in this case, integration of the two pathways causes a measurable phase delay (Nozue et al., 2007). We are interested in the multiple ways by which light and clock signals are integrated in the translation apparatus, where neither light regulation nor clock regulation are well characterized. Here we examined RPS6 phosphorylation in the presence and absence of a clock and in the presence and absence of light-dark cycles (LD versus LL). We found that alternation of light and darkness imposed a 12 ± 3 h shift in phase on the clock-driven cycle of RPS6-P, which is rather unusual. For comparison, the growth rate of the *Arabidopsis* root peaks around subjective dawn in wild-type under LL, i.e., under the control of the clock, but peaks at the end of day in the clock-deficient *elf3* strain under LD (Yazdanbakhsh et al., 2011). However, in this case, in wild-type under LD root growth peaks shortly after dawn. Thus, when the clock is operating in a light-dark cycle, the clock overrides the phase set by the light-dark cycle (Yazdanbakhsh et al., 2011). In summary, in this published example, the clock overrides the light-dark signals, whereas in the case of RPS6-phosphorylation, the light-dark cycle overrides the clock. In an attempt to find additional clock outputs that mimic the pattern set by RPS6 S237-P, we mined published data for transcripts with a day-peak in both WT in LD (Mockler et al., 2007) and CCA1-ox in LD (Missra et al., 2015), and a peak that was shifted by 12 ± 2 h in WT LL (Mockler et al., 2007). Surprisingly, there were less than a dozen (for examples, AT5G48880, AT1G16080, AT5G58770, AT5G01410, AT1G64770), and they did not share an obvious functional characteristic. Therefore, we conclude that the signal transduction that connects light and clock signals to RPS6-P is quite unusual.

### How Do Light and the Clock Interact to Drive Cycles of RPS6-P?

The interaction between light-dark signals and clock signals can be described as a quantitative computation of contrasting inputs (see Results; scenario iv). Although we cannot infer the precise mathematical form of the computation from the data, the simplest hypothesis states that light-dark and clock signals act independently, which predicts that the phosphorylation status follows the product rule. The independent action of clock and light signals on RPS6-P could have its biochemical basis in the activation of S6 kinase by two independent inputs (but see discussion below). For example, the mRNAs for S6K1 and S6K2 cycle with opposite phases, S6K1 peaking around dawn and S6K2 peaking around ZT16 (Mockler et al., 2007; Henriques et al., 2010). Alternatively, one could hypothesize that one input drives phosphorylation of RPS6, and the other input drives phosphatase activity. Outside of plants, RPS6 is dephosphorylated by a PP1 phosphatase, known as Glc7 in yeast, whose activity is subject to regulation (Belandia et al., 1994; Yerlikaya et al., 2016); the equivalent plant enzyme has not been characterized.

It can be considered whether the clock acts as a gate by regulating the light responsiveness of the RPS6 phosphorylation pathway. Under this scenario, during the subjective night, the RPS6-P would be highly sensitive to light while during subjective day, sensitivity would be decreased. If the gating activity of the clock were strong enough, this might explain why RPS6-P peaks during the subjective night in LL. However, some of our data are not easily reconciled with the gating hypothesis. For example, RPS6-P has a tendency to drop late in the light period in WT while it remains high in CCA1-ox (Supplementary Figures 4A–D), opposite to what is expected under the gating hypothesis.

### Does RPS6 Phosphorylation Reflect Activity of the TOR Signaling Pathway?

RPS6 is phosphorylated by the S6 kinase, which in turn is activated by TARGET OF RAPAMYCIN (TOR) kinase. There is abundant evidence that activation of TOR results in S6 phosphorylation in *Arabidopsis*. For example, treatment with the TOR kinase inhibitors Torin1 and AZD8055 will suppress S6 kinase phosphorylation and RPS6 phosphorylation, respectively (Schepetilnikov et al., 2013; Dobrenel et al., 2016). RPS6 phosphorylation is also a well established output of yeast and mammalian TOR activity (Biever et al., 2015; Yerlikaya et al., 2016). However, it cannot be ruled out that other kinases besides TOR regulate S6 kinase. In fact, S6 kinase is phosphorylated and activated by 3-phosphoinositide dependent kinase PDK1 (Turck et al., 2004) although the physiological regulators of PDK1 are not well characterized. If we assume that RPS6-P is governed by the TOR pathway under our conditions, then our data lead us to infer that TOR is activated by light through phytochrome and perhaps other photoreceptors. In keeping with this, stimulation of TOR activity by photoreceptor input was recently described (Pfeiffer et al., 2017). Clock signals might be integrated with the TOR pathway by either affecting TOR and S6 kinase activity or RPS6 phosphatase activity.

The pattern of RPS6-P in polysomes is also consistent with a model of the TOR signaling pathway. Activation of TOR, which activates S6 kinase and RPS6-P, was found to dislodge S6 kinase from polysomes, while inactive S6 kinase under resting conditions is polysome-associated (Schepetilnikov et al., 2013). This model is consistent with our data. If S6 kinase becomes activated while being released from polysomes, it should be present at a high concentration in the vicinity of polysomal RPS6. Hence activation of TOR by light or by return from heat to ambient temperature should preferentially phosphorylate the polysomal RPS6, which is what we observed. Maintaining the phosphorylation of RPS6 may not require a high concentration of S6 kinase and may therefore be achieved by free, non-polysomal S6 kinase. According to the model, under conditions when TOR becomes inactive, most likely in prolonged darkness and potentially after heat treatment, S6 kinase returns to an inactive state and becomes bound to polysomes, where it may be sterically hindered from maintaining the phosphorylation of polysomal RPS6 in the face of phosphatase activity, consistent with our data.

### Integration of Sucrose with Clock and Light-Dark Signaling

There is abundant evidence that photoassimilates, primarily sucrose, can modulate the function of the circadian clock, by entraining the clock (Haydon et al., 2013) and/or regulating the expression of central clock genes including PRR7 and GI (Dalchau et al., 2011; Muller et al., 2014; Shin et al., 2017; Shor et al., 2017). Sucrose is also known to interfere with light signal transduction (Dijkwel et al., 1997; Short, 1999). Sucrose also stimulates TOR kinase activity (Xiong and Sheen, 2014), and TOR/PDK1 – S6 kinase is the only kinase pathway known to drive RPS6 phosphorylation in *Arabidopsis* (Turck et al., 2004; Mahfouz et al., 2006; Dobrenel et al., 2016). Therefore, it seems plausible that supplementation of the growth medium with sucrose would interfere with the way that light-dark signals or clock signals regulate RPS6 phosphorylation, for example, by boosting the basal levels of RPS6 phosphorylation during the night or masking the drop in RPS6 phosphorylation during the dark period. Indeed, in etiolated 4-day-old seedlings, the presence of 1% sucrose in the growth medium strongly activated RPS6 phosphorylation to the degree that the induction of phosphorylation by light of various qualities was masked (**Figure 5**). However, in 12-day-old light grown seedlings the situation presented itself differently. The light-dark driven cycles of RPS6 phosphorylation seen in the clock-deficient CCA1-ox strain were not affected in any obvious way by a high concentration of sucrose. Likewise, the clock-driven cycle of RPS6-P S237 with a peak during the night could be seen at 0, 1, and 3% sucrose (Supplementary Figure 4), while the cycle for S240-P was weak under all sucrose conditions. Finally, the integration of light and clock signals in wild-type growing in a light-dark cycle was similar on 0 and 1% sucrose. At 3%, a high concentration, we observed variable results, with one of our replicates showing moderate day peaks and a second replicate showing clear night peaks in RPS6 phosphorylation at S237. It is therefore possible that a high concentration of sucrose has a tendency to confound the integration of light and clock signals.

The lack of a sucrose effect on the clock-driven cycle in constant light also speaks to the role of TOR kinase in RPS6-phosphorylation under these conditions. If one hypothesized that the clock-driven cycle in LL were mediated by a cycle of TOR activity, then the trough of phosphorylation during the day would be caused by low TOR activity. And given that TOR is stimulated by sucrose (Xiong and Sheen, 2014; Dobrenel et al., 2016), and RPS6-P was elevated by sucrose in constant darkness in our experiments, one would then have to predict that 1% and 3% sucrose would mask the trough of RPS6-P. However, this was clearly not the case (**Figure 6**). Therefore, the clock driven cycle of RPS6-P may not be caused by cyclical TOR activity, and TOR activity may be clock-independent. Testing this hypothesis will require additional experimentation.

Because the function of RPS6-P is unknown, it is unclear what benefit the plant may be deriving when the clock causes RPS6-P to oscillate with a peak at night. Night time is commonly associated with cooler temperatures, and RPS6-P is stimulated by cold. Therefore, it is tempting to consider that clock control of RPS6-P might be a rhythmic proxy for the diel oscillation in temperature, rather than light.

## Author Contributions

RE, SC, JT, OC, AR, and PA performed the experiments. RE, SC, RU-C, PA, and AvA analyzed the data. RE, SC, RH, and AvA designed the research. RE and AvA wrote the article.

## Conflict of Interest Statement

The authors declare that the research was conducted in the absence of any commercial or financial relationships that could be construed as a potential conflict of interest.
