# Supplementary material for: Phosphorylation of Ribosomal Protein RPS6 Integrates Light Signals and Circadian Clock Signals
Source: Front Plant Sci. 2018 Jan 19;8:2210. doi: 10.3389/fpls.2017.02210 (PMC5780430; doi:10.3389/fpls.2017.02210)
Supplement: Supplementary file 1 [file Presentation_1.PDF]

Supplementary Material

Phosphorylation of Ribosomal Protein RPS6 Integrates Light Signals and Circadian Clock Signals

Ramya Enganti [1)], Sung Ki Cho [1)], Jody D Toperzer [1)], Ricardo A Urquidi-Camacho [2)], Ozkan S Cakir [1)], Alexandria P Ray [1)], Paul E. Abraham [3)], Robert L. Hettich [2,3)] and Albrecht G von Arnim [*1,2)]

*** Corresponding author:** Albrecht G. von Arnim (vonarnim@utk.edu).

1 Supplementary Figures 1-5

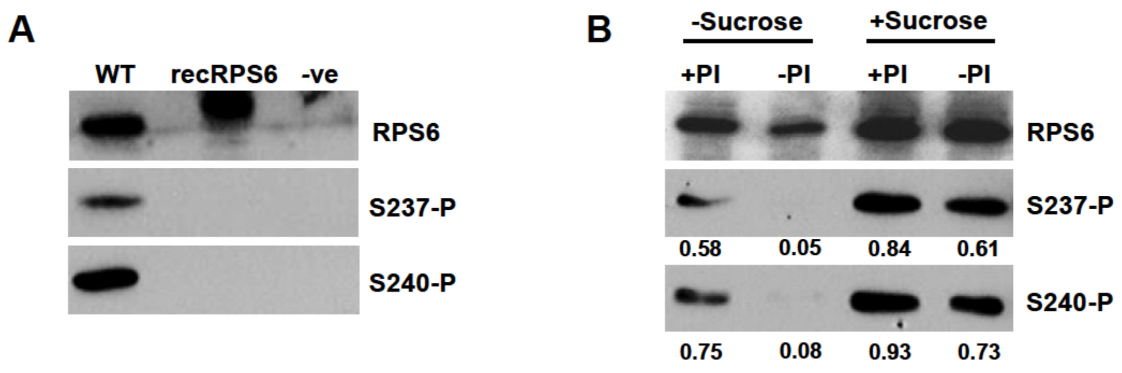

Supplemental Figure 1. **(A)** Phospho-specific antibodies do not recognize the unphosphorylated RPS6 protein. Western blot of (WT) wild-type *Arabidopsis* total cell extract and (recRPS6) recombinant His-tagged RPS6 protein produced in *E. coli*, and (-ve) His-tagged Nodulin 26 protein as a negative control probed with antibodies against RPS6, S237-P, and S240-P. **(B)** Phospho-specific antibodies recognize phosphorylated RPS6. Wild-type *Arabidopsis* seedlings were collected from plates without sucrose (lanes 1, 2) and with 1% sucrose (lanes 3, 4). Western blot of cell extracts prepared in the presence (+PI) and absence (-PI) of phosphatase inhibitors probed with the same antibodies as in (A). The numbers under the blots are ratios of S237-P/RPS6 in the middle panel and S240-P/RPS6 in the bottom panel.

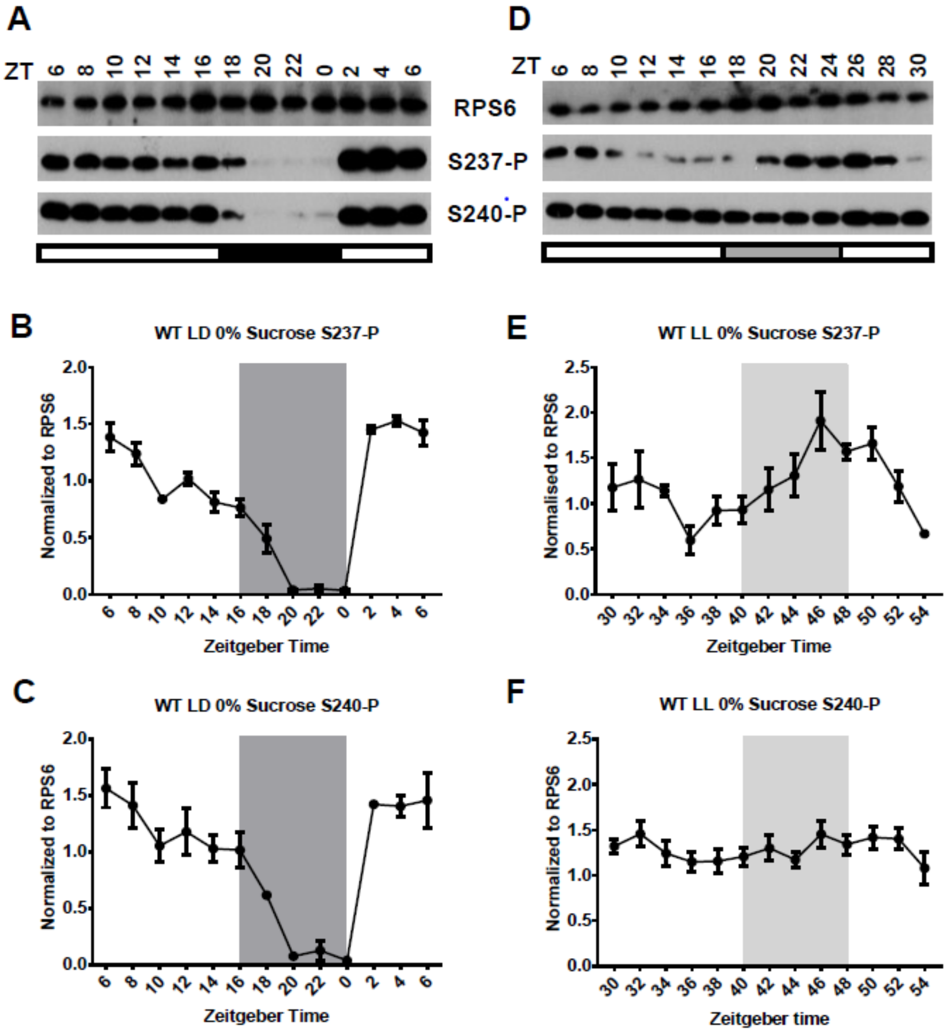

Supplemental Figure 2. RPS6-P cycles with a day peak under LD and with a night peak under LL conditions. Wild type seedlings were grown on medium lacking sucrose and entrained with long day conditions for 12 days and sampled every 2h for 24h starting at ZT6 **(A, B, C)** or entrained with LD for 11 days and shifted to continuous light for 1 day before sampling every 2h starting at ZT30 **(D, E, F)**. **(A, D)** Representative immunoblots for S237-P, S240-P and total RPS6. (**B, C, E, F**) Immunoblot signals for S237-P and S240-P were quantified and normalized against total RPS6 signals from the same time point, followed by median-centering each data series and averaging of the multiple replicates. Error bars show the standard error of the mean from n=2 replicates for LD and n=3 for LL.

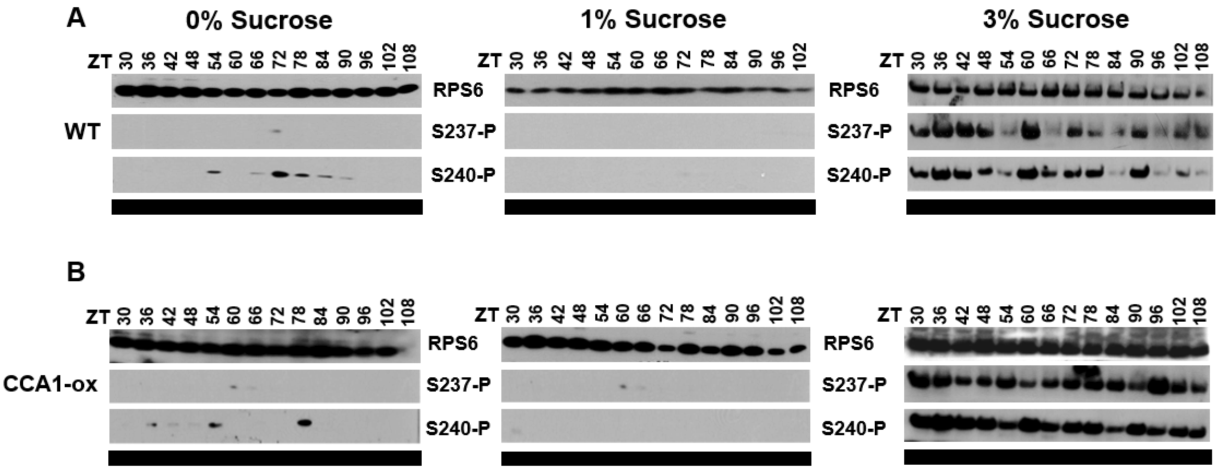

Supplemental Figure 3. Phosphorylation of RPS6 is nearly abolished or acyclic in continuous darkness. Twelve-day-old seedlings of **(A)** wild type and **(B)** CCA1-ox were grown for an additional four days in continuous darkness and RPS6-P scored by immunoblotting with phospho-specific antibodies against S237-P and S240-P. The left, middle, and right panels show plants grown in 0% sucrose, 1% sucrose, and 3% sucrose, respectively. Total RPS6 levels remained stable. For details see legend to **Figure 1**.

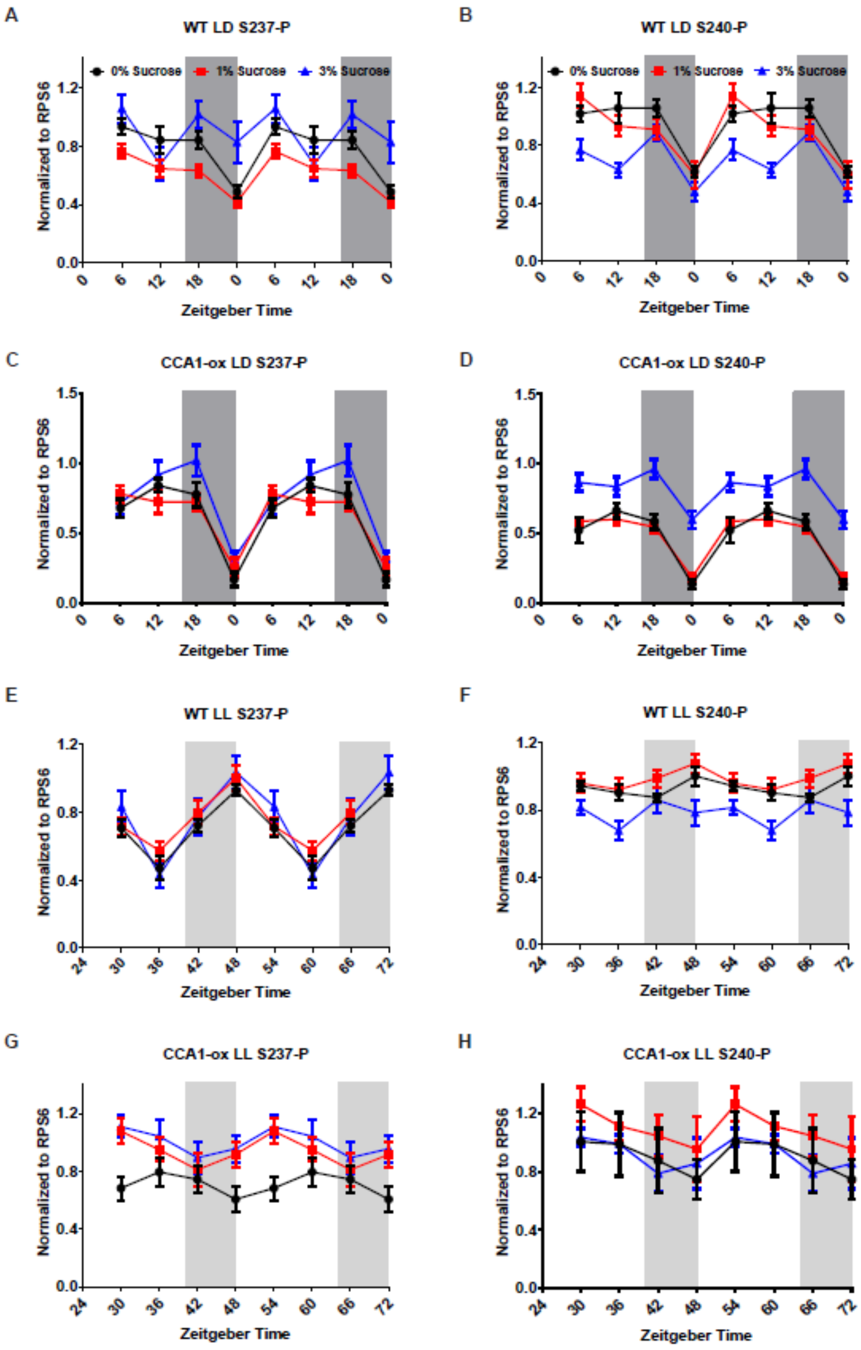

Supplemental Figure 4. RPS6 phosphorylation data from days 1-3 in Figures 1-4 and 6-7 were projected onto a single 24h time interval. The time interval was plotted twice in order to emphasize the cyclical nature of the data. **(A, B)** Wild type in a light-dark cycle. **(C, D)** CCA1-ox in a light-dark cycle. **(E, F)** Wild type in constant light. **(G, H)** CCA1-ox in constant light. Error bars display standard error of the mean for 3-4 replicates times 3 days (n=9-20). Note that the 1% sucrose traces (red) in (C, D) were median centered to the 0% sucrose traces (black) to adjust for a difference in exposure times of the immunoblots.

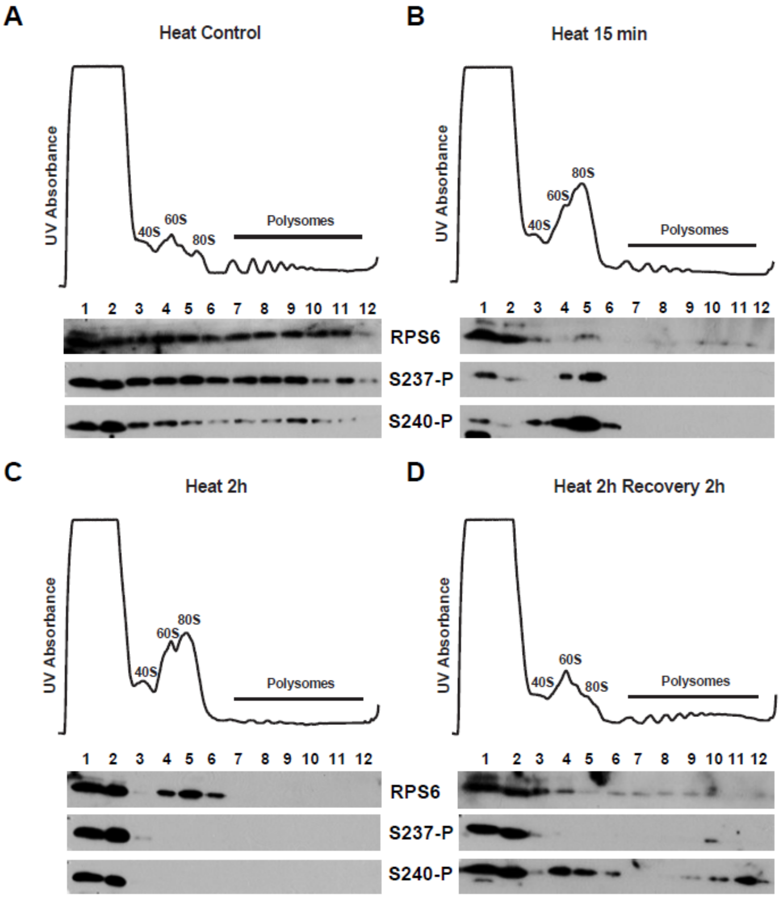

E

Heat (37°C)	Recovery (22°C)	P/(M+P)	Ratio	n
Control	-	0.52 ± 0.038	-	4
5 min	-	0.42 ± 0.003	0.81	2
15 min	-	0.29 ± 0.016	0.56	2
30 min	-	0.25 ± 0.010	0.48 ***	5
2h	-	0.24 ± 0.031	0.46 ***	4
2h	2h	0.47 ± 0.026	0.90	2
2h	6h	0.50 ± 0.011	0.96	2

Supplemental Figure 5. Heat stress causes a rapid decline in RPS6 phosphorylation in all ribosomal contexts, which recovers rapidly after return to ambient temperature. WT seedlings were grown on 0% sucrose under LD for 12 days and were subjected to no treatment **(A)** heat treatment for 15 minutes **(B)**, 2 hours **(C)** and allowed to recover for 2 hours after 2 hours of heat treatment **(D)**. The total cell extracts were subjected to sucrose gradient centrifugation and the fractions were probed for total RPS6 levels and phosphorylation at S237 and S240. **(E)** Table displaying the polysome/(monosome+polysome) (P/(M+P)) ratio of samples exposed to heat and recovery after heat treatment. A ratio of the P/(M+P) was calculated for all the treatments against the control. Standard deviation was calculated for the P/(M+P) ratios, n indicates number of biological replications, and *** indicates $p<0.001$ by Student's t-test.
